# Calcaneal Candida parapsilosis Osteomyelitis Causing Chronic Achilles Pain Following Achilles Corticosteroid Injection: A Case Report

**DOI:** 10.7759/cureus.92574

**Published:** 2025-09-17

**Authors:** Levi M Travis, Brandon Goldenberg, Steven D Steinlauf

**Affiliations:** 1 Department of Orthopaedics, University of Miami Miller School of Medicine, Miami, USA

**Keywords:** achilles tendon infection, ankle and foot, calcaneal osteomyelitis, candida parapsilosis infection, direct inoculation osteomyelitis, fungal osteomyelitis

## Abstract

Calcaneal osteomyelitis is a challenging condition to manage. It is associated with risk factors, such as diabetes, and develops from traumatic wounds or pressure ulcers in which microorganisms can spread contiguously to bone. Calcaneal osteomyelitis with *Candida parapsilosis* is exceptionally rare. *Candida* osteomyelitis of the foot and ankle is uncommon, and treatment strategies are often based on limited clinical experience. We report such a case of osteomyelitis from *Candida parapsilosis* causing chronic insertional Achilles pain in a 66-year-old nondiabetic female following an Achilles corticosteroid injection. *Candida parapsilosis* is a fungal species that typically exists as a commensal of human skin and has high rates of nosocomial spread through the formation of biofilms on medical instrumentation. This case highlights that even in immunocompetent patients with well-controlled chronic conditions, direct inoculation through corticosteroid injection can lead to *Candida* osteomyelitis and Achilles infection. Degenerative tissue may predispose a person to the infection. Cultures for bacteria and atypical organisms, along with pathologic interpretation, are crucial for diagnosis. Treatment with aggressive debridement and prolonged antifungal therapy, greater than six months, proved successful for *Candida* osteomyelitis of the calcaneus in this case.

## Introduction

Calcaneal osteomyelitis presents many challenges in its management. A major risk factor associated with the condition is diabetes, and it often evolves from open wounds, which allow for contiguous infectious spread [1,2]. Calcaneal osteomyelitis can also develop after open treatment and internal fixation of a fracture. The usual cause of calcaneal osteomyelitis is a bacterial infection, while a fungal infection is rare. *Candida* is often a concerning cause of infections, especially when infecting bone in the form of osteomyelitis, with ~9 cases of candidemia per 100,000 people per year in the United States from 2008-2016 [3,4]. *Candida* osteomyelitis can cause significant morbidity if not recognized early and treated appropriately. A minority of patients (~10%) have trauma or open wounds at the time of diagnosis [5]. Current literature describes only two cases of a specific type of calcaneal osteomyelitis due to *Candida parapsilosis*. One case describes a diabetic patient developing *Candida parapsilosis* osteomyelitis following intra-articular ankle corticosteroid injection, leading to septic arthritis [6]. Other reported cases of calcaneal *Candida* osteomyelitis are from the species *Candida albicans*, and are typically associated with diabetes mellitus [7-10]. We report a rare case of calcaneal osteomyelitis from *Candida parapsilosis* causing chronic insertional Achilles tendon pain in a 66-year-old, immunocompetent, nondiabetic female following an Achilles tendon corticosteroid injection. The novelty of this case stems from the lack of obvious clinical signs of osteomyelitis presenting in an immunocompetent patient and the gross appearance of tissue examination in the surgical field.

## Case presentation

**Figure 1:**
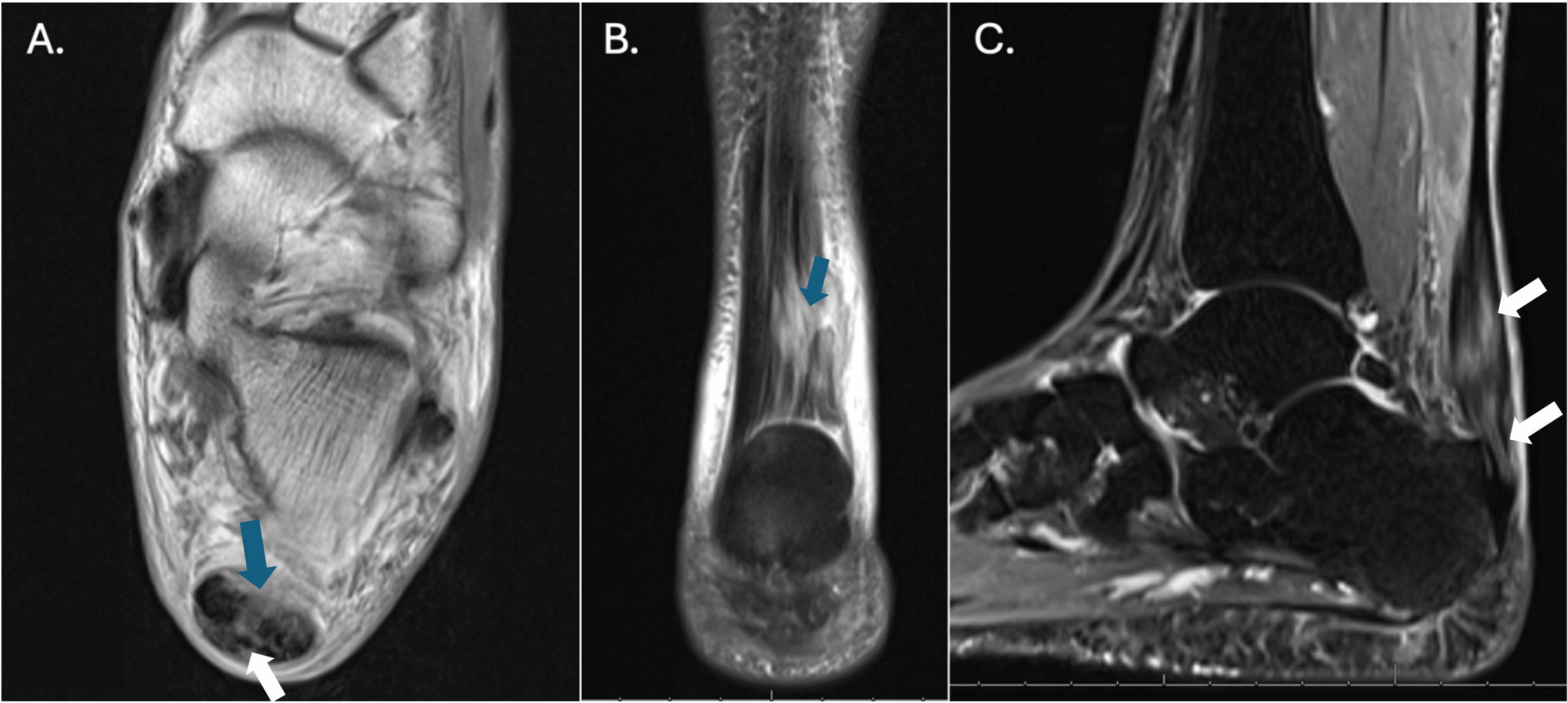
Repeat MRI in June 2022 demonstrating partial tearing (arrows) and significant tendinosis of the Achilles tendon A. Axial MRI image showing tendinosis and partial tearing (blue and white arrows) of the Achilles tendon fibers near the calcaneal insertion. B. Coronal image depicting thickened and heterogeneous Achilles tendon fibers with partial tearing (blue arrow). C. Sagittal T2-weighted image demonstrating abnormal signal intensity consistent with tendinosis and partial tearing (white arrows) at the Achilles tendon insertion.

A 66-year-old female with past medical history of treated Graves’ disease with resultant hypothyroidism and osteoporosis presented in 05/2023 with 29 months of left insertional Achilles tendon pain. The pain was first noted in December 2020 while doing home exercise during the COVID-19 pandemic. The patient never sustained trauma. Shortly after the onset of symptoms, she was placed into a walking boot for five weeks, followed by physical therapy. Three months of physical therapy provided no relief. MRI was obtained in March of 2021. It demonstrated a posterior ankle effusion, minimal retrocalcaneal bursitis, and minimal Achilles tendon degeneration. She received a single corticosteroid injection in March 2021, following the first MRI, which yielded no relief. A second period of immobilization followed by physical therapy proved unsuccessful. The following year, in 06/2022, a repeat MRI demonstrated a partial tear and significant tendinosis of the Achilles tendon (Figure 1).

On the day of presentation to our clinic in 05/2023, she complained of pain at the Achilles tendon insertion as well as lateral ankle numbness and swelling. She denied any radiculopathy. Her pain was worsened by activity, flat shoes, and walking barefoot. Pain was alleviated with rest and heeled shoes. Physical exam was significant for tenderness at the medial and lateral Achilles tendon insertion points. There was nodular thickening 2 cm above the Achilles tendon insertion and a small palpable gap just above the insertion. She denied any trauma or changes in her symptoms over the previous two years after the injection. Lateral radiographic imaging at this visit demonstrated two small calcifications. An MRI was not repeated secondary to the lack of change in symptoms.

In 08/2023, the patient underwent surgery for Achilles tendon debridement with possible repair. The subcutaneous tissues were atrophic, and there was a chronic rupture of the central 80% of the Achilles tendon. The remaining medial and lateral Achilles tendon insertions were significantly degenerative. The decision was made to remove the entire distal Achilles tendon with debridement of degenerative tissue. The posterior tuberosity appeared normal on the surface. The most proximal posterior tuberosity was resected, and a cyst, approximately 5 mm × 5 mm, was found in the lateral aspect of the tuberosity. The cyst contained soft tissue, which showed no obvious signs of infection. The cyst and resected Achilles tendon were sent for culture and pathology out of an abundance of caution. A peroneus longus allograft was utilized to reconstruct the Achilles tendon, along with a short harvest flexor hallucis longus tendon transfer.

**Figure 2:**
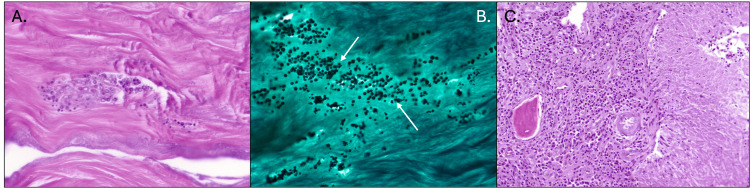
Histopathologic intraoperative findings (August 2023) of fungal tendon infection and associated osteomyelitis A. Hematoxylin and eosin (H&E) stain of necrotic Achilles tendon fibers, demonstrating degenerated collagen matrix and inflammatory cell infiltration. B. Gomori methenamine silver (GMS) stain showing numerous fungal organisms (arrows) consistent with *Candida parapsilosis* within the necrotic tendon matrix. C. H&E stain demonstrating granulomatous osteomyelitis in the adjacent bone, with multinucleated giant cells and chronic inflammatory infiltrate surrounding fungal elements.

All culture and pathology results postoperatively revealed *Candida parapsilosis* growth in the Achilles tendon, Achilles tendon insertion site, and the removed bone cyst (Figure 2). The patient continued to have no fevers, chills, or systemic symptoms of infection. The decision was made to remove the allograft, the interference screw holding the flexor hallucis longus, and any devitalized tissue. Anti-fungal-loaded beads were inserted. Fluconazole 200 mg PO daily was started as recommended by the Infectious Disease specialist. This was followed by Fluconazole 400 mg PO daily for a total of seven months.

**Figure 3:**
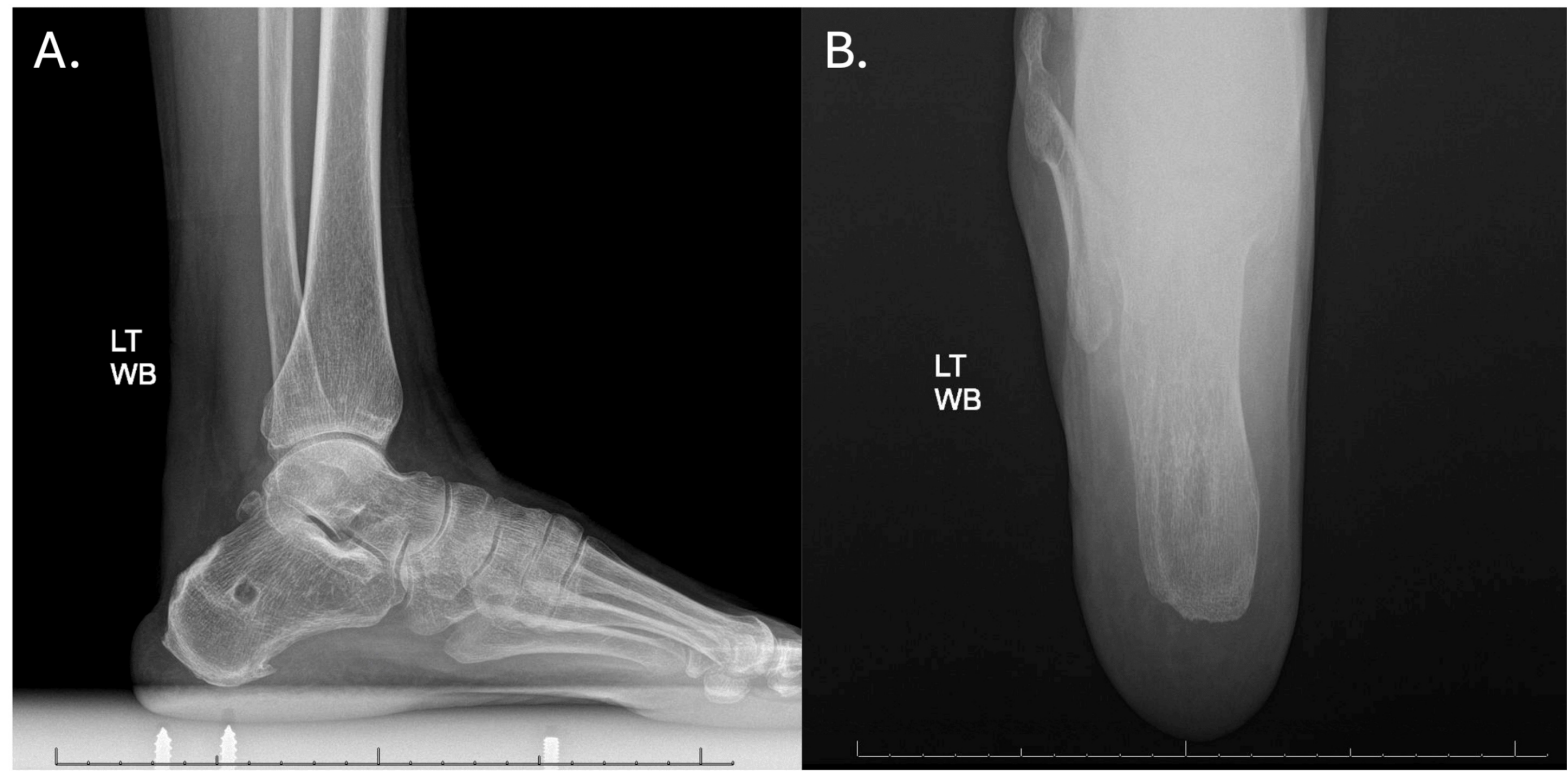
Follow-up radiographs (March 2025) of the left calcaneus (A) Lateral and (B) axial weight-bearing radiographs demonstrate surgical changes status post Achilles tendon reconstruction and calcaneal debridement posterosuperiorly with no evidence of recurrent infection or osteolysis.

At one month follow-up after the Achilles tendon allograft removal and debridement, she continued to lack any signs of clinical infection. Her tenuous soft tissue envelope took two months to heal. She was kept in an equinus splint with limited movement. Once the wounds were healed, therapy was begun for range of motion and strengthening. The decision was made not to attempt further reconstruction unless necessary. At six-month follow-up, the patient reported stiffness in the Achilles tendon, but had returned to weightlifting, biking, and Zumba, along with physical therapy twice per week. At the eight-month follow-up, she finished the course of Fluconazole and had no complaints. On physical exam, the Achilles tendon region had filled with scar tissue, was nontender, and her wounds remained healed. She had symmetrical resting tension, range of motion, and 5/5 plantar flexion strength. She had resumed full activity. She remains asymptomatic with normal function at one-year follow-up after the debridement surgery. Figure 3 shows radiographs at the patient's most recent follow-up appointment, in March 2025, approximately 19 months postoperatively.

## Discussion

In this case, the chronic localized Achilles tendon pain, intraoperative degenerative findings, and positive culture and histopathology together confirmed the diagnosis of *Candida parapsilosis* osteomyelitis. *Candida* osteomyelitis is typically characterized by a chronic course with variations in the severity of presenting and long-term symptoms. Due to the lower frequency of *Candida* osteomyelitis, especially in foot and ankle infections, there are few comprehensive analyses, and evidence-based treatment is guided by smaller sample-sized reports [5]. Diagnosis of *Candida* osteomyelitis with a preceding episode of candidemia usually has a delay of diagnosis from one week up to several months due to its insidious onset, with less than half of patients having a fever; further, erythrocyte sedimentation rate (ESR), C-reactive protein (CRP), and white blood cell (WBC) count is often minimally elevated [5]. Localizing symptoms are most commonly present in chronic forms, with 90% of patients with *Candida* osteomyelitis having localizing pain, tenderness, and/or edema [5]. A high index of suspicion should be maintained in cases of current or prior candidemia and risk factors for *Candida* infection, including antibiotic use, immunosuppression, including the use of corticosteroids, and forms of subcutaneous penetration such as central venous catheter, injection drug use, and trauma or surgery [11,12].

Shorter courses of antifungal agents of approximately three months have demonstrated increased rates of infection relapse, and so extending treatment to six to 12 months may be more effective [13]. Further, one must consider the unique difficulties of treating osteomyelitis in the calcaneus, Achilles tendon insertion, and Achilles tendon. Management of calcaneus osteomyelitis requires excision of necrotic bone and soft tissue, dead space management, preservation of weight-bearing capability, and good soft tissue coverage, with less radical surgery being favored [1]. While there are few reports of *Candida* osteomyelitis, there are fewer discussing *Candida parapsilosis*, as it was the cause of only 7% of *Candida* osteomyelitis cases in a case series of 207 cases of *Candida *osteomyelitis [5]. *Candida parapsilosis* is a fungal species that exists in a yeast phase or pseudohyphal form, is typically a commensal of human skin, and has high rates of nosocomial spread through formation of biofilms on medical instrumentation [14]. *Candida parapsilosis* typically infects joint spaces after procedures that are invasive or involve prosthetic devices [14,15]. In the cases of *Candida parapsilosis* osteomyelitis that exist in the literature, most are associated with patients who have suppressed immune systems or other risk factors for fungal infections, such as HIV, cirrhosis, cancer, diabetes, prolonged corticosteroid use, alcoholism, or renal failure [16,17].

Following initial work-up of acute onset of pain and three months of conservative treatment, the patient received a corticosteroid injection, followed by 18 months of chronic insertional Achilles tendon pain. At the time of surgical treatment to repair the Achilles tendon, there was little suspicion of osteomyelitis. The patient did not experience or present with fever, chills, erythema, or significant swelling; her primary symptoms consisted of pain and tenderness. No inflammatory markers were checked secondary to the lack of signs of infection. The fungal infection was discovered through surgical culture and pathologic evaluation of a small cyst in the calcaneus and was managed with repeat surgery and antifungal pharmacotherapy. Given the discovery of the Achilles tendon partial tear, it is unclear if her symptoms were caused by the chronic infection or steroid-induced tendon tearing. However, it still remains that infection was soon diagnosed and a full course of anti-fungal treatment resolved the patient’s symptoms.

Though the patient was immunocompetent, the patient’s history of hypothyroidism may have decreased her immunity. Hypothyroidism and low levels of T4 and T3 are associated with a bidirectional diminishing humoral and immune cell response, potentially contributing to an increased risk of fungal infection [18,19]. However, this patient’s hypothyroidism was well-controlled at the time of infection with a free T4 and thyroid-stimulating hormone (TSH) of 1.60 and 1.65, respectively, making direct inoculation through corticosteroid injection three months after her injury the most likely cause of her calcaneus osteomyelitis and Achilles tendon infection. Previously damaged joints and soft tissue are predisposed to *Candida parapsilosis* infection following skin barrier opening or injection, such as with corticosteroids [20]. The degenerative change of this patient’s Achilles tendon may have predisposed her to fungal seeding and chronic infection. Further, as *Candida parapsilosis* is a commensal human skin organism, it is mostly transmitted through direct inoculation. In the 2012 analysis of 207 *Candida* osteomyelitis cases, direct inoculation accounted for 25% of cases, while hematogenous and contiguous infection accounted for 67% and 9% of cases, respectively [5]. The proposed mechanism by which the calcaneus was infected is through direct inoculation into the Achilles tendon insertion during injection, followed by contiguous spread along the Achilles tendon, as the species was cultured at the tendon, the tendon insertion, and in the calcaneus bone itself. While this infection was most likely from skin flora, instances of osteoarticular infections as a result of contaminated corticosteroids have occurred. These instances of corticosteroid contamination by fungal species include the methylprednisolone contamination of 2012, which led to 751 cases of fungal meningitis, stroke, and spinal and osteoarticular infections, and 64 deaths [21]. Short time intervals between corticosteroid injections and excessive dose administration are also associated with a higher risk of complications such as infection [22].

This case of calcaneus osteomyelitis from *Candida parapsilosis* was successfully managed through removal of the previously placed allograft and hardware and debridement of the calcaneus bone and Achilles tendon to remove potentially compromised tissue. The decision to use an aggressive approach of extended antifungal therapy for seven months, with fluconazole 400 mg, has been shown to decrease the risk of infectious relapse and has been successful in preventing clinical symptoms in this patient up to the present at 12-month follow-up [5,13].

## Conclusions

This case report underscores the need for heightened vigilance in diagnosing subclinical fungal osteomyelitis in patients with chronic pain and elevated inflammatory markers. Further, it demonstrates the importance of maintaining a sterile field during major and minor procedures, such as outpatient injections. This case highlights that even in immunocompetent patients, direct inoculation through corticosteroid injection can lead to *Candida* osteomyelitis and Achilles tendon infection, with previously damaged tissue potentially further predisposing the patient to seeding. Cultures for not only bacteria but also atypical organisms and pathologic interpretation are crucial for diagnosis. Treatment with aggressive debridement and prolonged antifungal therapy, greater than six months, proved successful so far for *Candida* osteomyelitis of the calcaneus in this case.
